# CDCA8 Contributes to the Development and Progression of Thyroid Cancer through Regulating CDK1

**DOI:** 10.7150/jca.64747

**Published:** 2022-04-18

**Authors:** Cheng Xiang, Wu-hui Sun, You Ke, Xing Yu, Yong Wang

**Affiliations:** 1 Department of Thyroid Surgery, The Second Affiliated Hospital of Zhejiang University College of Medicine, Hangzhou, Zhejiang, China.; 2 Department of Nephrology, The Second Affiliated Hospital of Zhejiang University College of Medicine, Hangzhou, Zhejiang, China.

**Keywords:** thyroid cancer, CDCA8, CDK1, cell proliferation, cell apoptosis

## Abstract

**Background**: This study aims to reveal regulatory role of cell division cycle associated 8 (CDCA8) in thyroid cancer progression and metastasis.

**Methods**: A series of experiments *in vivo* and *in vitro* were performed to explore the function of CDCA8 in thyroid cancer.

**Results**: Immunohistochemical analysis showed that CDCA8 expression levels were upregulated in thyroid cancer tissues compared with normal tissues, and were statistically correlated with tumor stage. Results of *in vitro* loss-of-function assay showed that downregulation of endogenous expression of CDCA8 could significantly inhibit cell proliferation, colony formation, cell migration, and promote apoptosis. Thyroid cancer cells lacking CDCA8 expression also had reduced tumorigenicity *in vivo*. Further, results of preliminary mechanistic exploration showed that CDK1 may be a potential downstream molecule of CDCA8 in regulating thyroid cancer progression. We subsequently confirmed that CDK1 itself exerted a significant regulatory function in thyroid cancer by loss- and gain-of-function experiments. Moreover, overexpression of CDK1 could weaken the tumor suppressive effect caused by CDCA8 knockdown.

**Conclusions**: CDCA8 functions as an oncogene in thyroid cancer, and CDCA8 knockdown suppresses cancer development *in vitro* and *in vivo*. Additionally, CDK1 was further identified as a potential target of CDCA8 in thyroid cancer.

## Introduction

Thyroid cancer is the most common endocrine system tumor in the world, and its incidence is still increasing rapidly year by year 1, 2. At present, the treatment of thyroid cancer mainly includes surgery, radiotherapy, endocrine therapy, chemotherapy and targeted treatment. Although the 10-year survival rate of patients with thyroid cancer has reached more than 90% after surgery combined with ^131^I radiotherapy and L-thyroxine suppression therapy, there are still some thyroid cancer patients showing metastasis in the early stage of the disease or weak absorption of ^131^I, resulting in poor prognosis 3. It is well known that the occurrence and development of tumors are composed of complex factors and steps, and large number of molecules participate in many of the regulatory steps. Therefore, molecular targeted therapy which recognizes specific molecular target of thyroid cancer is of great importance for improving prognosis, which is an effective supplement to the traditional treatment 4-6. However, due to the largely unclear molecular mechanism of thyroid cancer, currently the molecular targeted drugs approved for thyroid cancer treatment are mostly multi-target drugs, such as sorafenib, lovatinib and sunitinib 7, 8. This is only a drop in the bucket for the need to overcome thyroid cancer. Therefore, it has become one of the most popular research hotspots to seek for new molecules involved in the regulation of tumor occurrence and development and develop corresponding targeted therapeutic drugs 9, 10.

Chromosomal passenger complex (CPC) is a key regulatory factor in the process of cell mitosis. Through the mutual regulation of 4 core proteins and precise positioning in time and space, it guarantees the normal progress of mitosis *via* the correction of kinetochore microtubule connection, the activation of spindle assembly and the initiation of cytoplasmic division 11, 12. The overexpression of CPC component protein can lead to the disorder of CPC function, as well as the repair and regulation in mitosis, and cause abnormal cell division and aneuploidy formation, thus promoting the occurrence and development of malignant tumors 13, 14. Increasing evidence has indicated that Aurora kinase B (Aurora B) and Survivin (baculoviral IAP repeat containing 5, BIRC5) are abnormally overexpressed in a variety of tumors, and specific inhibitors targeting them have shown promising antitumor activity in clinical trials 15-17. Another CPC component, cell division cycle associated 8 (CDCA8, alias Borealin/DasraB), has been found to be involved in the development of breast cancer, cutaneous melanoma and bladder cancer 18-21. In addition, the study has verified NF-Y as a positive regulator of CDCA8 transcription, which unearthed the underlying activation of CDCA8 expression in embryonic stem cells and cancer cells 22. In terms of thyroid cancer, previous study has initially identified CDCA8 as a key biomarker in anaplastic thyroid cancer 23. However, the exploration on the role of CDCA8 in the development and metastasis of thyroid cancer remains to be insufficient.

In this study, our results delineated the significant upregulation of CDCA8 in thyroid cancer tissues, which may lead to fast progression thus advanced tumor stage of thyroid cancer patients. The *in vitro* fundamental assessment of CDCA8 manifested that CDCA8 may act as a tumor promotor in thyroid cancer through regulating cell proliferation, formation of colonies, cell apoptosis and cell migration, the validation of which was also confirmed by mice model with transplanted tumor. Moreover, the subsequent mechanism research showed that CDCA8 regulated the expression of CDK1 thus influencing the development of thyroid cancer. Therefore, it was supposed in this study that CDCA8/CDK1 signaling may have substantial contribution in the development and progression of thyroid cancer.

## Material and Method

### Cell culture

B-CPAP, TPC-1 and CAL-62 cell lines of human thyroid carcinoma were obtained from BeNa Technology (Hangzhou). B-CPAP and TPC-1 were cultured in CM1-1 culture medium containing 90% DMEM-H and 10% FBS, and CAL-62 were maintained in complete medium containing 90% DMEM (Invitrogen, #12430) and 10% FBS.

### Samples collection and immunohistochemistry (IHC)

Totally, 40 patients with papillary thyroid cancer who underwent surgical treatment were enrolled in this study obtained from Xi'an Alenabio Co., Ltd. (#TH801a), with the informed content collected. The patients enrolled in this study have no history of surgery and other malignant tumors. Besides, none of the patients was suffering from pre-operative managements, such as chemotherapy or radiotherapy. The samples were finally diagnosed thyroid cancer by post-operative pathological examinations. At the same time, the patients with non-papillary thyroid cancer and these subjects with thyroid-related diseases such as Hashimoto's thyroiditis were excluded. Samples of the paracarcinoma thyroid tissue (approximately 10 mm distance from the tumor) of 40 patients were used as normal group. All sample tissues were obtained at the time of initial surgery, frozen in liquid nitrogen, and stored at -80°C.

For IHC, briefly, the sections were baked in an oven at 60°C for 30 min, then the sections were dewaxed and rehydrated, immersed into citrate buffer for in 3 min at 98°C for antigen retrieval, blocked with 3% hydrogen peroxide and 5% BSA. These sections were then incubated with primary antibodies and followed incubated with appropriate secondary antibodies. Sections were visualized with DAB solution and counterstained with hematoxylin. Pictures were captured by microscopic and analyzed by ImageScope and CaseViewer. The antibodies were listed in Table S1.

### Lentiviral vector construction, Plasmids and transfections

Primer amplification sequence of human CDK1 gene and RNAi sequences of CDCA8 (RNAi-105340) were designed in Shanghai Bioscienceres, Co., Ltd. Sequences were detailed in Table S2. Related overexpression lentivirus vector and interference lentivirus vector were constructed and qualified plasmids were obtained with EndoFree Maxi Plasmid Kit (QIAGEN, #Y5-12381) and used for packaging. Cells were transfected using Lipofectamine 2000 reagent (Invitrogen) according to the manufacturer's instructions and incubated at 37 °C. After cultured for 72 h, cell infection efficiency was evaluated by microscopic fluorescence.

### RT-qPCR

Total RNA from transfected B-CPAP and TPC-1 cells was extracted by TRIzol reagent (Shenggong, #B610409-0100) and was qualified using Nanodrop 2000/2000C spectrophotometer (Thermo Fisher Scientific) according to the manufacturer's instructions. 2.0 μg RNA was reverse transcribed into cDNA using Promega M-MLV kit (Heidelberg). RT-qPCR was performed with SYBR Green mastermixs Kit (Vazyme, #Q111-03), the relative quantitative analysis in gene expression data were analyzed by the 2^-ΔΔCt^ method. Primer sequences of upstream and downstream was showed in Table S3.

### Western blot analysis

Total protein from transfected B-CPAP and TPC-1 cells was extracted by ice-cold RIPA buffer (Servicebio, # G2002). The same amount of total protein from each cell group (20 μg) was separated by 10% SDS-PAGE (Invitrogen) and electrotransferred onto a PVDF membrane (Bio-Rad Laboratory). The membrane was blocked in TBST plus 5% non-fat milk, and incubated with primary antibodies and appropriate secondary antibody. Antibodies were showed in Table S1. The signals were visualized using enhanced chemiluminescence reagents (ECL, Bio-Rad) and protein band intensities were quantified using Image J software (NIH).

### Co-Immunoprecipitation (Co-IP) assay

Cells were lysed by pre-ice-cooled IP lysate, collected in EP tube and centrifuged at 13,000×g. Total protein was collected and concentrated by BCA. 1.2 mg total protein in the tube was incubated with antibody overnight. 20 μL beads were added and centrifuged at 1000×g, and then the incubated protein was added for incubating for 1-2 h at 4°C. After centrifuged at 2000 ×g and washed, IP lysate and appropriate 5× loading buffer were added and incubated at 100°C for 5 min. Proteins were separated by 10% SDS-PAGE electrophoresis and transferred into PVDF membrane. The membrane was blocked with 5% skimmed milk for 1 h and incubated with primary antibodies overnight and continuing incubated with corresponding secondary antibody. After washing with TBST, the blots were visualized by enhanced chemiluminescence (ECL, Amersham).

### MTT assay

Cell viability of lentivirus infected B-CPAP and TPC-1 cells was measured by MTT assay. Exponential growth phase cells were seeded onto 96-well plates (3,000 cells/well). 20 μL of MTT solution (5 mg/mL, Gongsheng, # A600799-0250) was added and incubated for 4 h. The absorbance values at 490 nm and 570 nm were measured by microplate reader (Tecan) at 24 to 120 h after cell seeding. The cell viability ratio was calculated as cell viability (%) = optical density (OD) treated/OD control × 100%.

### Cell apoptosis

Cell apoptosis evaluation was performed by flow cytometry method using Annexin V-APC (eBioscience, # 88-8007-72) according to the manufacturer's protocol. Cells were seeded into 6-well plate for 5 days and 10 μL Annexin V-APC (eBioscience) was added and incubated at room temperature without light for 15 min, 300μL binding buffer was added for measuring using FACSCalibur (BD Biosciences).

### Cell growth assay

Lentivirus transfected B-CPAP and TPC-1 logarithmic growth phase cells were cultured in 96-well plates (2,000 cell/well) for 5 days. Cell numbers were counted every day with a Celigo Imaging Cytometer (Nexcelom Bioscience) and the 5-day cell proliferation curve was drawn.

### Colony formation assay

1,000 transfected B-CPAP and TPC-1 cells were seeded into 6-well plates and further cultured until the cell number of most individual clones reaches 50 or more. The culture medium was exchanged every 3 days. The clones were photographed with a fluorescence microscope (Olympus). All clones were fixed and stained by 4% paraformaldehyde (Sinopharm Chemical Reagent) and Giemsa (Shanghai Dingguo, # AR-0752), respectively. Stained colons were photographed and colony forming rate was calculated.

### Wound healing assay

Lentivirus transfected B-CPAP and TPC-1 cells (5×10^4^ cells/well) were plated into a 96-well dish and cultured until the confluence of cells reached 90%. A 96-wounding replicator (VP scientific) was used for cell scratch making and debris were gently washed away. After 4 h and 16 h culturing, photographs of cell scratches were taken and cell migration rates were calculated.

### Transwell migration assay

Transwell migration assay was performed as followed: B-CPAP and TPC-1 cells transfected with CDCA8 knockdown vector and CDK1 knockdown or overexpression vector with their respective vector controls, were seeded in the upper chambers (Corning) of 24-well plate (5×10^4^ cells/well) in 100 μL/well medium without FBS. The lower chambers were filled with 600 μL/well medium supplemented with 30% FBS. Cells were incubated for 24 h at 37°C and then non-metastatic cells were removed. Cells were fixed by 4% formaldehyde and stained by Giemsa. The migration ability of cells was analyzed.

### Genechip analysis

Total RNA was extracted using TRIZOL Reagent (Life technologies) following the manufacturer's instructions and checked for a RIN number to inspect RNA integrity by an Agilent Bioanalyzer 2100 (Agilent technologies). Total RNA was amplified, labeled and purified by using GeneChip 3'IVT PLUS Reagent Kit (# 901838, Affymetrix) followed the manufacturer's instructions and scanned by GeneChip® Scanner 3000 (Affymetrix). Raw data were normalized by RMA algorithm in R. Significant difference analysis and functional analysis based on Ingenuity Pathway Analysis (IPA) (Qiagen) was executed, and |Z - score| > 2 is considered meaningful.

### *In vivo* tumorigenicity assay

4 × 10^7^ shCDCA8 and shCtrl lentivirus transfected logarithmic growth phase CAL-62 cells were subcutaneous injected into each female BALB/c nude mice (4-week-old, n = 10) which were purchased from Shanghai Lingchang Animal Technology Co., Ltd. for *in vivo* tumorigenicity. Tumor size and weight was recorded two times per week. Fluorescence imaging by IVIS Spectrum Imaging System (Perkin Elmer) were performed after mice were anesthetized by intraperitoneal injection of Pentobarbital Sodium (0.7%, 10 μL/g). Next, all anesthetized mice were sacrificed and tumors were removed. Tumor tissues were prepared for Ki-67 immunostaining assay to detect the expression lever of histone Ki-67. The animal study was taken placed in Clinical research center of The Second Affiliated Hospital of Zhejiang University College of Medicine, and reviewed and approved by Institutional Animal Care and Use Committee of The Second Affiliated Hospital of Zhejiang University College of Medicine.

### Bioinformatics analysis

In order to verify the clinical significance of CDCA8 in thyroid cancer, we evaluated the influence of CDCA8 expression on the 10-year relapse free survival of patients through Kaplan-Meier plotter database (http://www.kmplot.com/). We also used Gepia database (http://gepia.cancer-pku.cn/) to analyze the correlation between CDCA8 expression and tumor stage in thyroid cancer. Subsequently, we detected 20 significant genes associated with CDCA8 used GeneMINIA database (https://genemania.org/), and then explored their related biological process using BinGO plug-in of Cytoscape. *P*<0.001 was set as the significant level.

### Statistical Analysis

Data are expressed as the mean ± SD. Student's t-Test was used to analyze the statistical significance between two groups, and ANOVA was used to identify and analyze the differences among three groups or more. The IHC values were compared using the Mann-Whitney U analysis. All statistical analysis was performed using SPSS 17.0 (IBM) and GraphPad Prism 6.01 (GraphPad Software) and *P*<0.05 was considered statistically significant.

## Results

### Identification of CDCA8 as potential promoter in thyroid cancer

**Figure 1:**
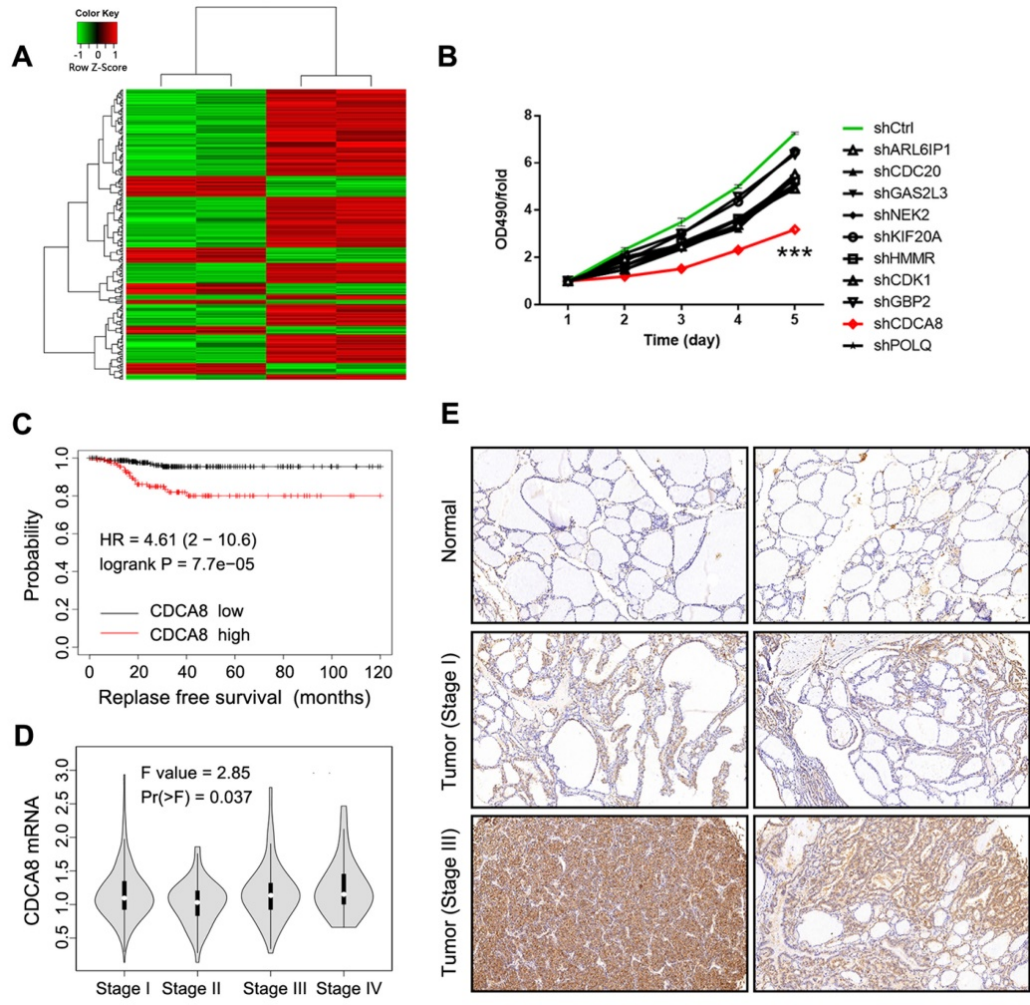
** Identification of CDCA8 as potential promoter in thyroid cancer.** (A) RNA sequencing was performed to screen differentially expressed genes between thyroid cancer tissues and normal tissues (2 v 2). (B) The capability of the knockdown of various significantly upregulated candidates to inhibit thyroid cancer cell growth was evaluated through MTT assay. (C) The expression of CDCA8 in normal tissues and thyroid cancer tissues with different pathological stage was detected by IHC. The data was shown as mean with SD. *P<0.05, **P<0.01, ***P<0.001

In this study, gene microarray technology was used to study and explore the molecular mechanism of thyroid cancer. The whole gene expression profiles of thyroid cancer clinical tissues and corresponding normal tissues were obtained and compared. In general, we screened the data with multiple change ≥ 2 or ≤ 0.5 and *P <* 0.05 from gene expression profile, and identified 298 differentially expressed genes, including 80 up-regulated genes and 218 down-regulated genes (Figure 1A). In order to search for tumor promoters in thyroid cancer, we selected 10 genes that were significantly up-regulated in B-CPAP cells, including ARL6IP1, CDC20, GAS2L3, NEK2, KIF20A, HMMR, CDCA8, CDK1, POLQ, and GBP2 (Figure S1A). The multiple changes of these genes were the highest, and the lentiviral plasmid expressing the corresponding shRNA was constructed. The lentiviral plasmids were transfected into B-CPAP cells respectively, and their inhibitory effects on cell growth were detected by MTT assay. As shown in Figure 1B, shCDCA8 plasmid presented the highest inhibition rate and significantly inhibited cell proliferation after transfection (*P*<0.001). Therefore, CDCA8 was identified as the research focus in our next experiments. Survival analysis indicated an unfavorable impact of CDCA8 high expression on the relapse free survival of patients (*P* < 0.001, Figure 1C). Further analysis found the correlation between CDCA8 expression and cancer stage (*P* = 0.037, Figure 1D), and CDCA8 higher expression was observed in patients with advanced stage. These results initially revealed the clinical significance of CDCA8 in thyroid cancer. Subsequently, we verified the expression of CDCA8 in 40 tumor samples and their paracarcinoma thyroid tissue, and immunohistochemistry analysis showed that the expression of CDCA8 in thyroid cancer tissues was significantly increased (Figure 1E, Table 1). But we found no significant correlation between CDCA8 expression and tumor stage among cancer patients (Table 2), which possibly due to the insufficient sample size as the *P* value close to significant level. The above results suggested that CDCA8 might exacerbate the development of thyroid cancer.

### Knockdown of CDCA8 inhibits proliferation, colony formation, migration, and promotes apoptosis of thyroid cancer cells

**Figure 2:**
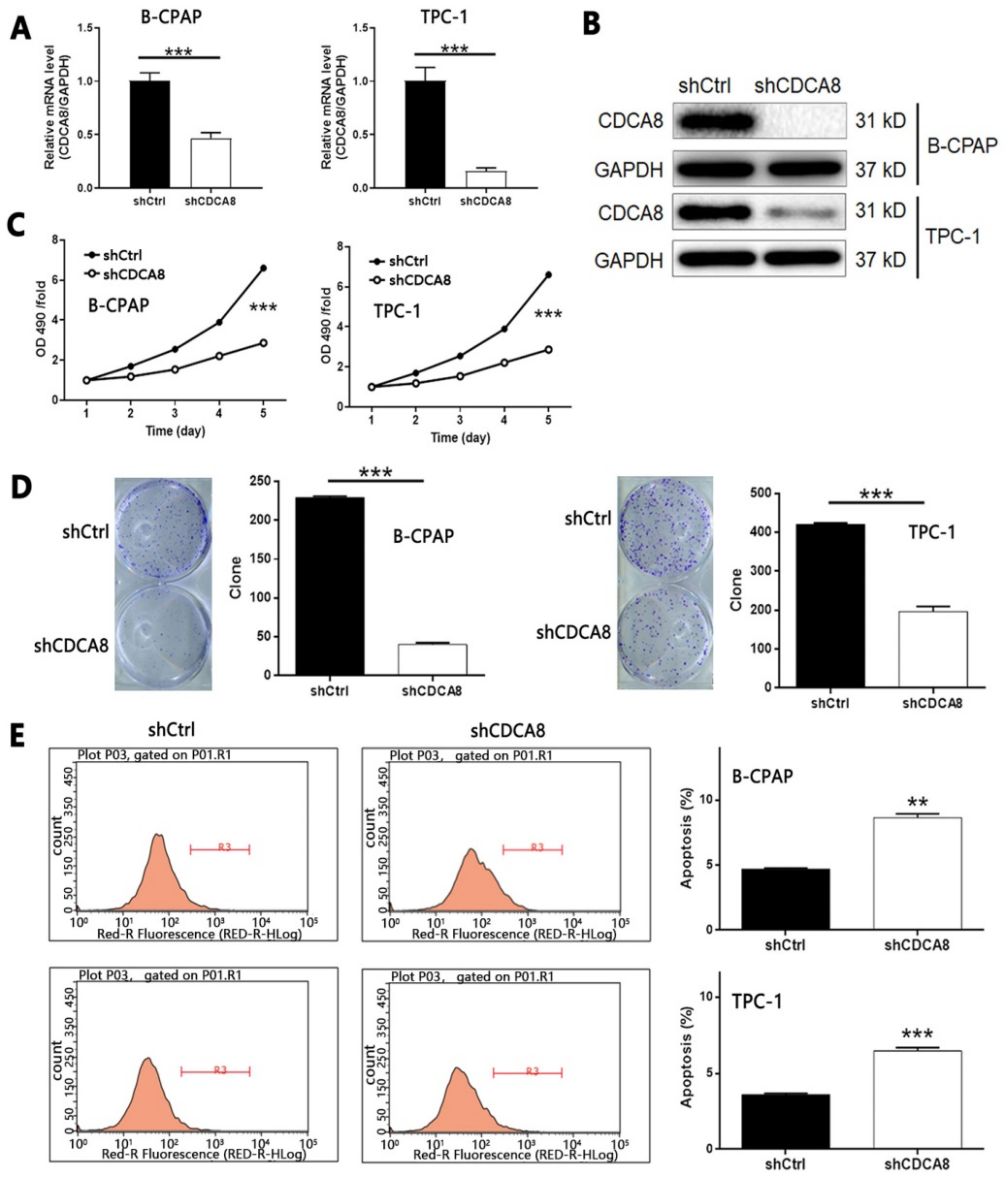
** Knockdown of CDCA8 affects the proliferation, colony formation and apoptosis of TC cells.** The qPCR (A) and western blotting (B) were used to verify the construction of CDCA8 knockdown cell models. (C) The effects of CDCA8 knockdown on cell proliferation was examined by MTT assay. (D) Colony formation assay was performed to reveal the effects of CDCA8 on cell growth. (E) Flow cytometry was utilized to visualize the impacts of CDCA8 on cell apoptosis. The data was shown as mean with SD. *P<0.05, **P<0.01, ***P<0.001

Considering the up-regulation of CDCA8 in thyroid carcinoma, we used B-CPAP and TPC-1 cells transfected with shCDCA8 lentiviral vector to detect cell function, and shCtrl transfected cells were used as negative control. The transfection efficiency was detected by fluorescence imaging (> 80%, Figure S1B). B-CPAP cells were used to screen shRNA with the highest silencing efficiency on CDCA8. Among the three candidates, RNAi-10540 was the most efficient, so RNAi-10540 was used in all subsequent experiments (*P* < 0.01, Figure S1C). The knockdown efficiency of CDCA8 in B-CPAP and TPC-1 cells was evaluated by qPCR (all *P* < 0.001, Figure 2A) and Western blotting (Figure 2B), indicating that CDCA8 knockdown cell model was successfully constructed. MTT assay was used to detect the effect of CDCA8 on cell proliferation. Compared with shCtrl group, the cell growth of shCDCA8 group was significantly inhibited (all *P* < 0.001, Figure 2C). Similar results were obtained in colony formation test. Compared with shCtrl group, the colony count of shCDCA8 group was significantly reduced (all *P* < 0.001, Figure 2D). In addition, compared with shCtrl transfected cells, CDCA8 knockdown cells showed a higher percentage of apoptotic cells (*P* < 0.01 in B-CPAP cells and *P* < 0.001 in TPC-1 cells, Figure 2E). The results of human apoptosis antibody array showed that knockdown of CDCA8 may induce apoptosis by regulating Bax, caspase 3, CD40, CD40L, Fas, FasL, p21, p27, survivin and XIAP (Figure S2A). In addition, we evaluated the changes of Akt, p-Akt, CCND1, CDK4 and Cdk6 expression in TPC-1 cells after CDCA8 depletion to elucidate the regulatory mechanism (Figure S2B).

**Figure 3:**
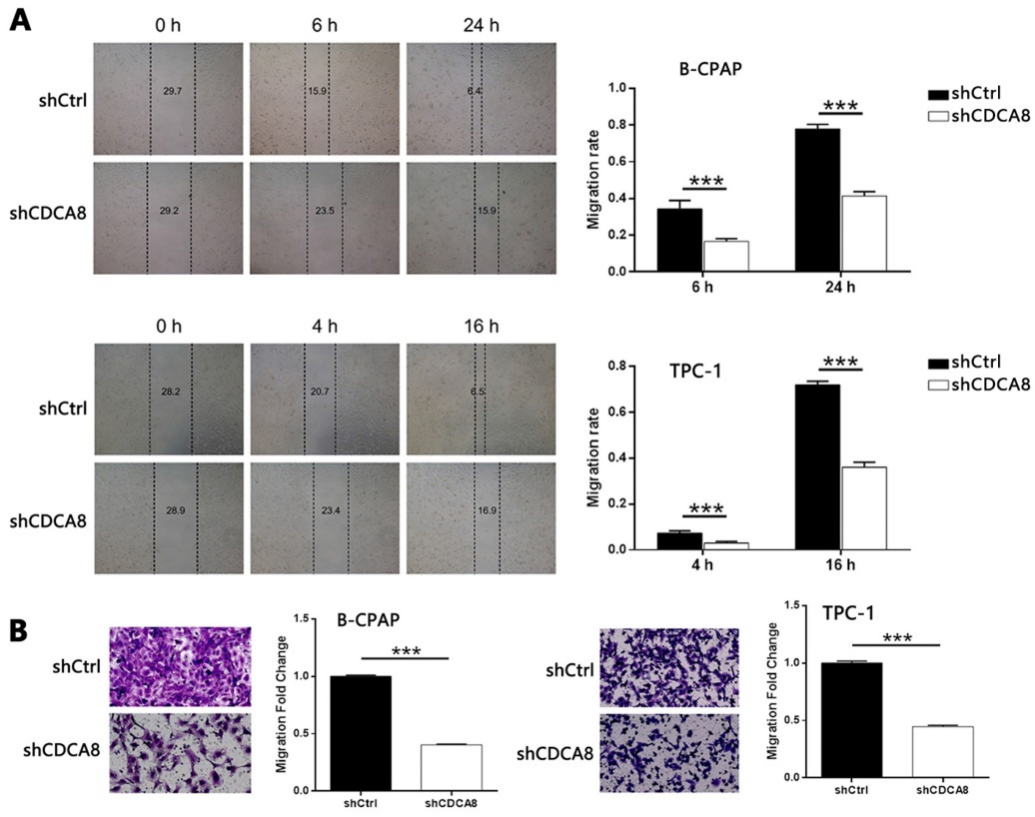
** Knockdown of CDCA8 affects the migration and invasion of TC cells.** Migration of cells with or without CDCA8 knockdown was detected by wound-healing assay (A) and Trans-well assay (B). The representative image was randomly selected from at least 3 independent experiments. The data was shown as mean with SD. *P<0.05, **P<0.01, ***P<0.001

Subsequently, wound healing and Trans-well tests were used to evaluate the effect of CDCA8 on the migration of thyroid cancer cells. As shown in Figure 3A-3B, CDCA8 knockdown significantly inhibited the migration of B-CPAP and TPC-1 cells (all *P* < 0.001). These results showed that the proliferation, migration, and apoptosis of thyroid cancer cells were significantly inhibited by down regulating CDCA8.

### Knockdown of CDCA8 inhibits tumor growth of thyroid cancer *in vivo*

**Figure 4:**
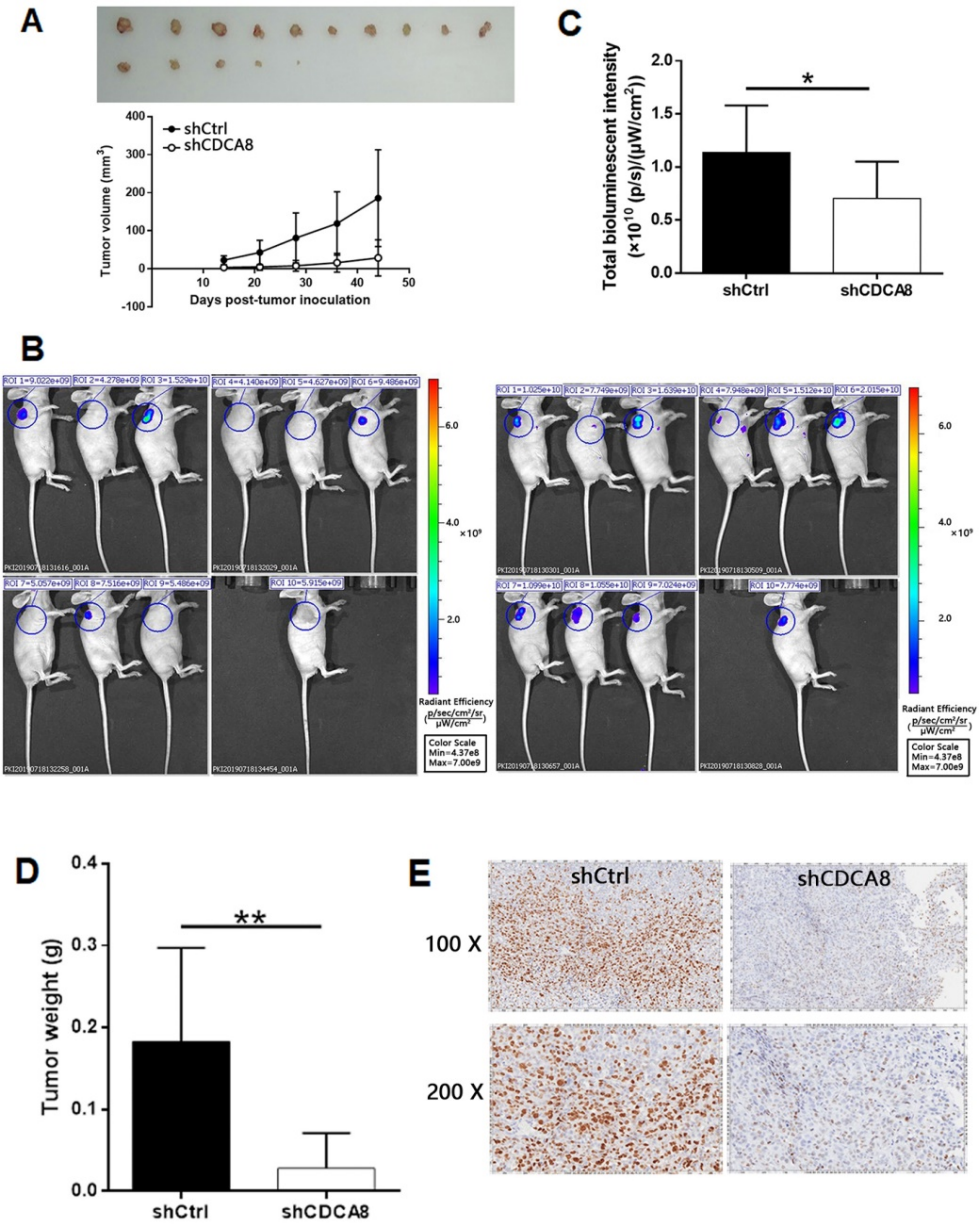
** CDCA8 knockdown inhibits thyroid cancer development *in vivo*.** (A) The measurement of tumor volume indicated that tumors formed by cells with absence of CDCA8 grew much slower. Inset: photo of the removed tumors. (B) The *in vivo* bioluminescence imaging of xenograft mice models showed the much weaker bioluminescence intensity in shCDCA8 group of mice. (C) The intensity of bioluminescence in mice was scanned and used to represent the tumor burden. (D, E) After sacrificing the mice, tumors were removed and collected for weighting and detecting Ki-67 expression by IHC. The data was shown as mean with SD. *P<0.05, **P<0.01, ***P<0.001

In order to further study the effect of CDCA8 on the growth of thyroid cancer, a mouse xenograft model was established. Two groups of mice were intravenously injected with CDCA8 knockdown or non-knockdown CAL-62 cells. We observed that compared with shCtrl group, mice injected with CDCA8 knockdown CAL-62 cells had smaller tumor volume, slower growth rate and therefore smaller tumor size (Figure 4A). Before the end of the experiment, we conducted* in vivo* fluorescence imaging experiments and weighed the tumor 44 days after injection to verify the inhibitory effect of CDCA8 knockdown on tumor growth (Figure 4B-4D). In addition, IHC analysis showed that tumors in shCDCA8 group showed significantly lower expression of Ki-67 (a representative factor of tumor growth) (Figure 4E). These results suggested that CDCA8 knockdown can inhibit the growth of thyroid cancer.

### CDCA8 may promote development of thyroid cancer *via* regulation of CDK1

**Figure 5:**
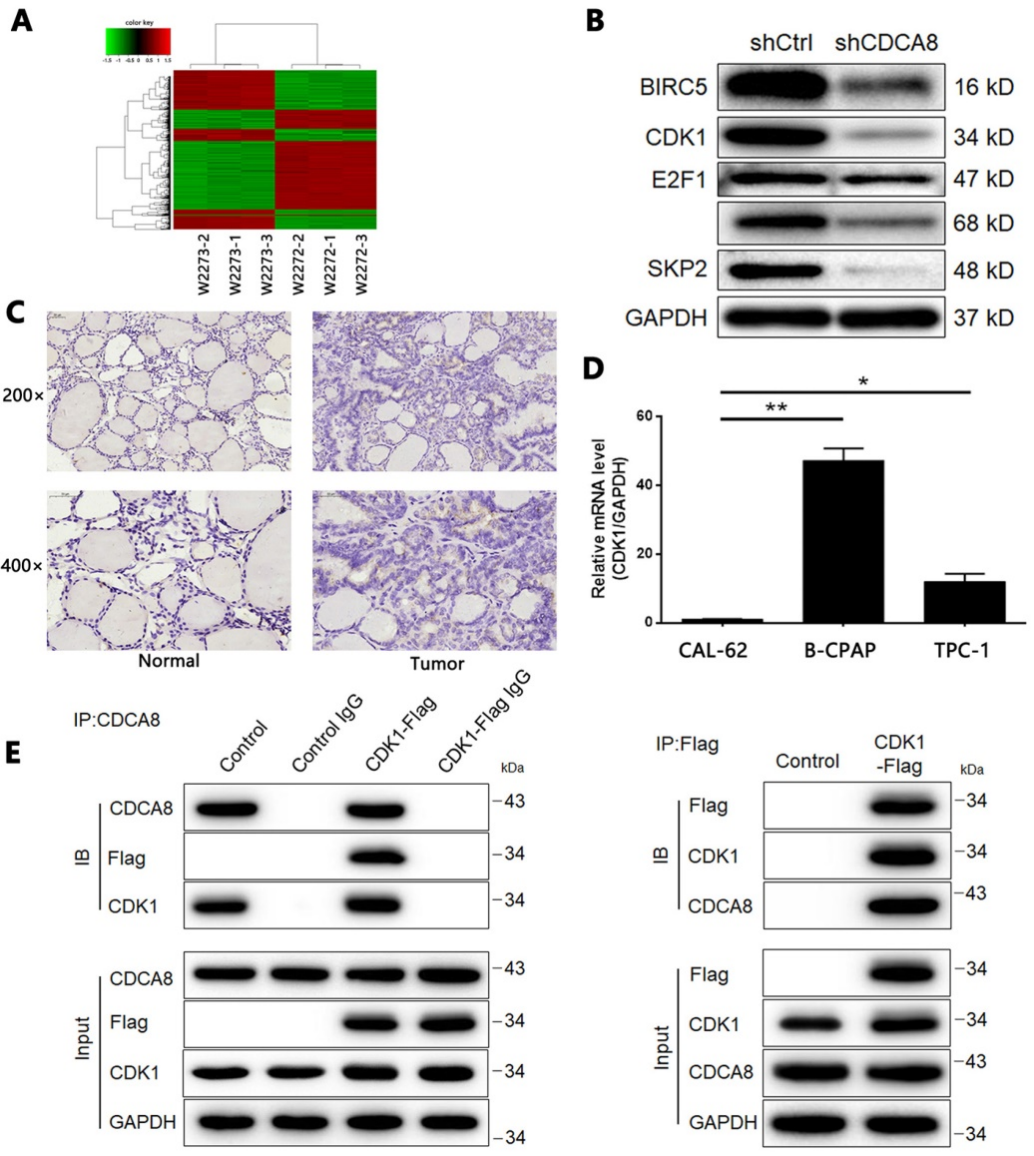
** Screening of potential downstream target of CDCA8 in thyroid cancer.** (A) RNA sequencing was performed to screen differentially expressed genes between cells in shCtrl and shCDCA8 groups (3 v 3). (B) The expression of several downregulated DEGs was verified by western blotting in TPC-1 cells. (C) The expression of CDK1 in normal tissues and thyroid cancer tissues was detected by IHC. (D) The endogenous expression of CDK1 in thyroid cancer cell lines including CAL-62, B-CPAP and TPC-1 was detected by qPCR. (E) The direct interaction between CDCA8 and CDK1 was examined by co-immunoprecipitation. The data was shown as mean with SD. *P<0.05, **P<0.01, ***P<0.001.

According to the preliminarily functional analysis on CDCA8, we found that CDCA8 significantly participated in cell cycle-related biological processes, such as M phase of mitotic cell cycle, nuclear division, and chromosome segregation (Figure S3). We then used RNA-seq analysis to analyze the gene expression profile of TPC-1 cells transfected with shCtrl or shCDCA8, so as to further explore the downstream mechanism of CDCA8 in regulating thyroid cancer. Using the same screening threshold, 2553 differentially expressed genes (DEGs) were identified, including 1132 up-regulated genes and 1421 down regulated genes (Figure 5A, Figure S4A-4B). In addition, IPA technology was used to analyze the classic pathway and disease and function of DEGs (Figure S4C-4D). The qPCR (Figure S4E) and Western blotting (Figure 5B) were used to verify the expression changes of several groups of genes in DEGs in TPC-1 cells. In view of the enrichment of DEGs in cyclins and cell cycle regulatory signaling pathways, we speculated that CDCA8 may promote the development of thyroid cancer by upregulating CDK1. It should be noted that CDK1 was also identified as an up-regulated DEG in thyroid cancer tissues in the above RNA sequencing. The protein expression level of CDK1 in tumor tissues was higher than that in normal tissues (Figure 5C), which was consistent with the sequencing results. Background detection of endogenous CDK1 expression in thyroid cancer cell line also showed that CDK1 had higher expression in B-CPAP and TPC-1 cells compared with another thyroid cancer cell line CAL-62 (Figure 5D). More importantly, in the co-IP experiment, we observed a direct interaction between CDCA8 and CDK1 (Figure 5E).

### Overexpression/Knockdown of CDK1 regulates proliferation, colony formation, apoptosis and migration of thyroid cancer cells

**Figure 6:**
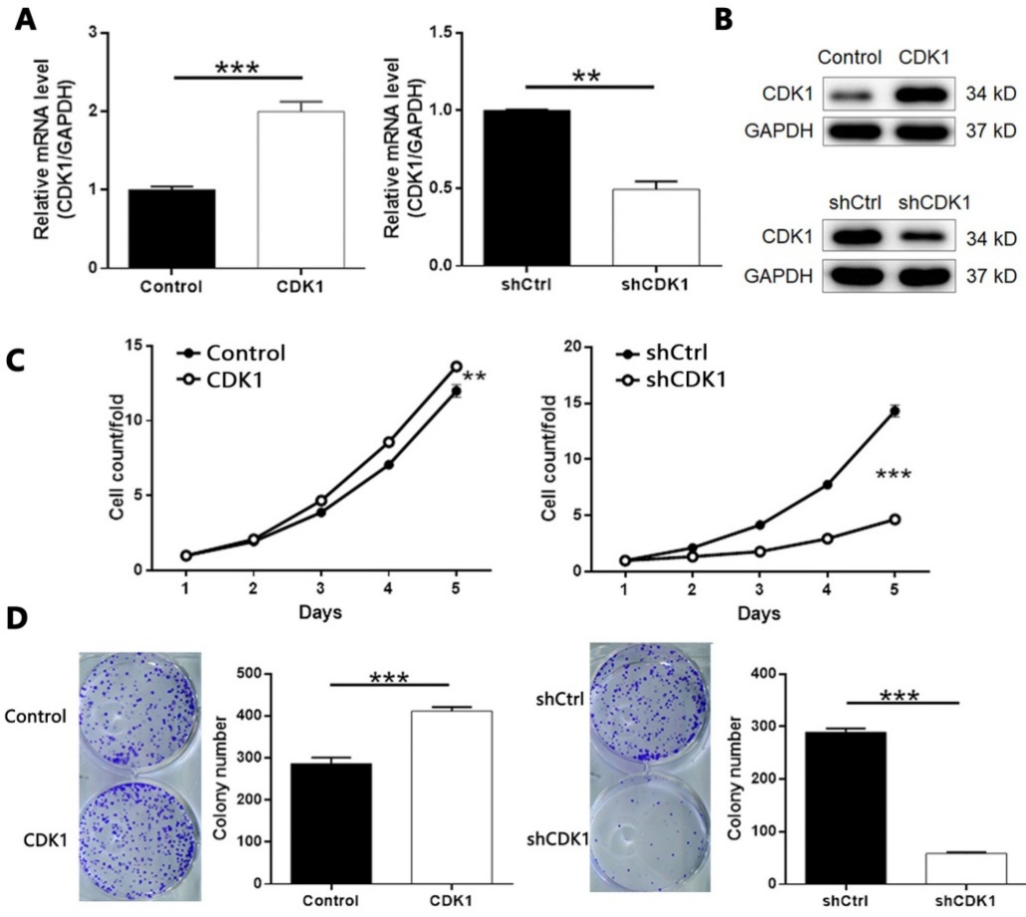
** The role of CDK1 in thyroid cancer.** The qPCR (A) and western blotting (B) were performed to verify the knockdown or overexpression of CDK1 in TPC-1 cells. (C) The effects of CDCA8 on cell proliferation was examined by MTT assay. (D) Colony formation assay was performed to reveal the effects of CDCA8 on cell growth. The data was shown as mean with SD. *P<0.05, **P<0.01, ***P<0.001.

Based on the above experiments, we designed three shRNAs targeting CDK1 to screen effective CDK1 knockdown targets. As shown in Figure S5, shCDK1-1 showed the highest knockdown efficiency (about 88%) detected by qPCR, so we applied it to all subsequent experiments. At the same time, we used similar methods to artificially increase the endogenous expression of CDK1 in TPC-1 cells to construct a cell model of CDK1 overexpression. After confirming the transfection efficiency (Figure S6), we further verified the overexpression and knockdown effects of CDK1 in TPC-1 cells by qPCR and Western blotting (Figure 6A-6B). As expected, MTT assay showed that CDK1 overexpression promoted the proliferation of thyroid cancer cells, and vice versa (Figure 6C, CDK1 overexpression *P* < 0.01, CDK1 knockdown *P* < 0.001). Similar results were obtained in the plate clone formation experiment (Figure 6D,* P* < 0.001).

**Figure 7:**
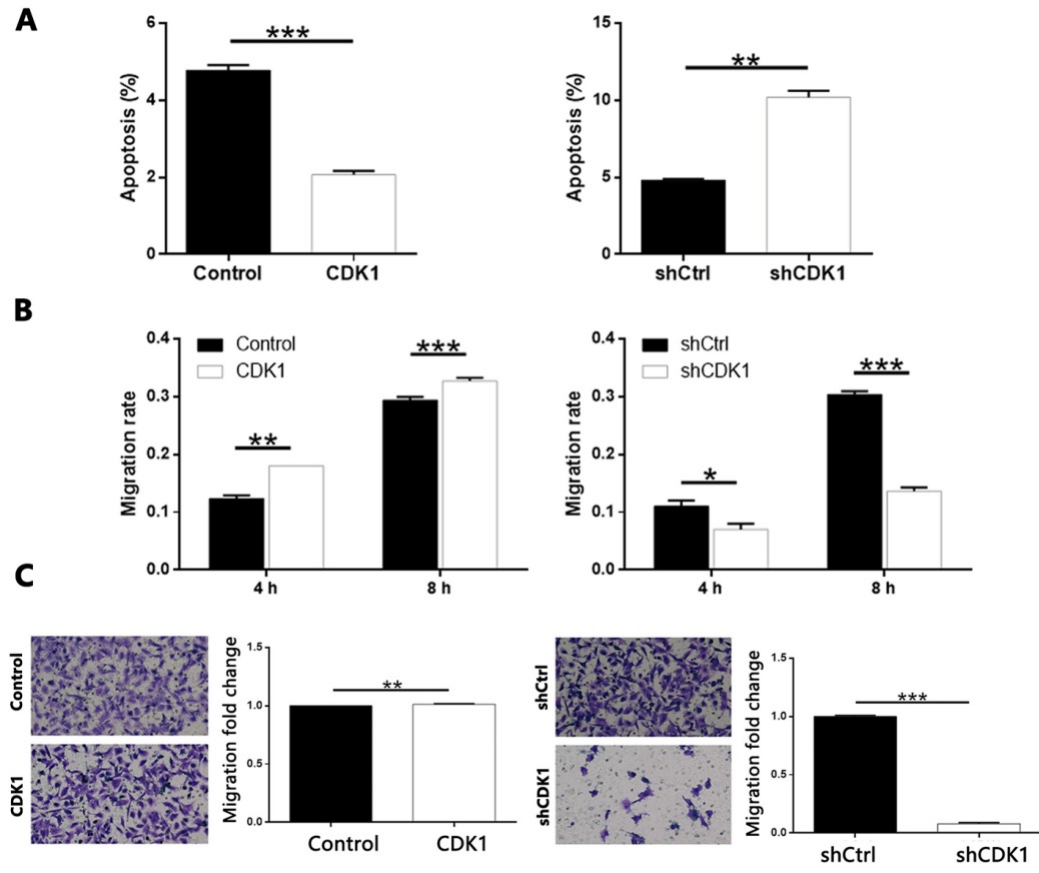
** The role of CDK1 in thyroid cancer.** (A) Flow cytometry was utilized to visualize the impacts of CDCA8 on cell apoptosis. Migration of cells with or without CDCA8 knockdown was detected by wound-healing assay (B) and Transwell assay (C). The representative image was randomly selected from at least 3 independent experiments. The data was shown as mean with SD. *P<0.05, **P<0.01, ***P<0.001.

In addition, overexpression of CDK1 inhibited the apoptosis of thyroid cancer cells, and CDK1 knockdown caused apoptosis promotion of cancer cells (Figure 7A, CDK1 overexpression *P* < 0.001, CDK1 knockdown* P* < 0.01). On the other hand, through cell scratch test (Figure S7 and Figure 7B, *P* < 0.001 at 8 h) and Transwell analysis (Figure 7C, *P* < 0.001), we concluded that result was similar with that of CDCA8 knockdown. The decrease of CDK1 endogenous expression also significantly inhibited the cell growth of thyroid cancer cells, and results from CDK1 overexpression model were opposite (Figure 7B, *P* < 0.001; Figure 7C, *P* < 0.01). Therefore, these results suggest that CDK1 plays a key role in the development of thyroid cancer.

### Knockdown of CDCA8 impairs the promotion of thyroid cancer by CDK1 overexpression

**Figure 8:**
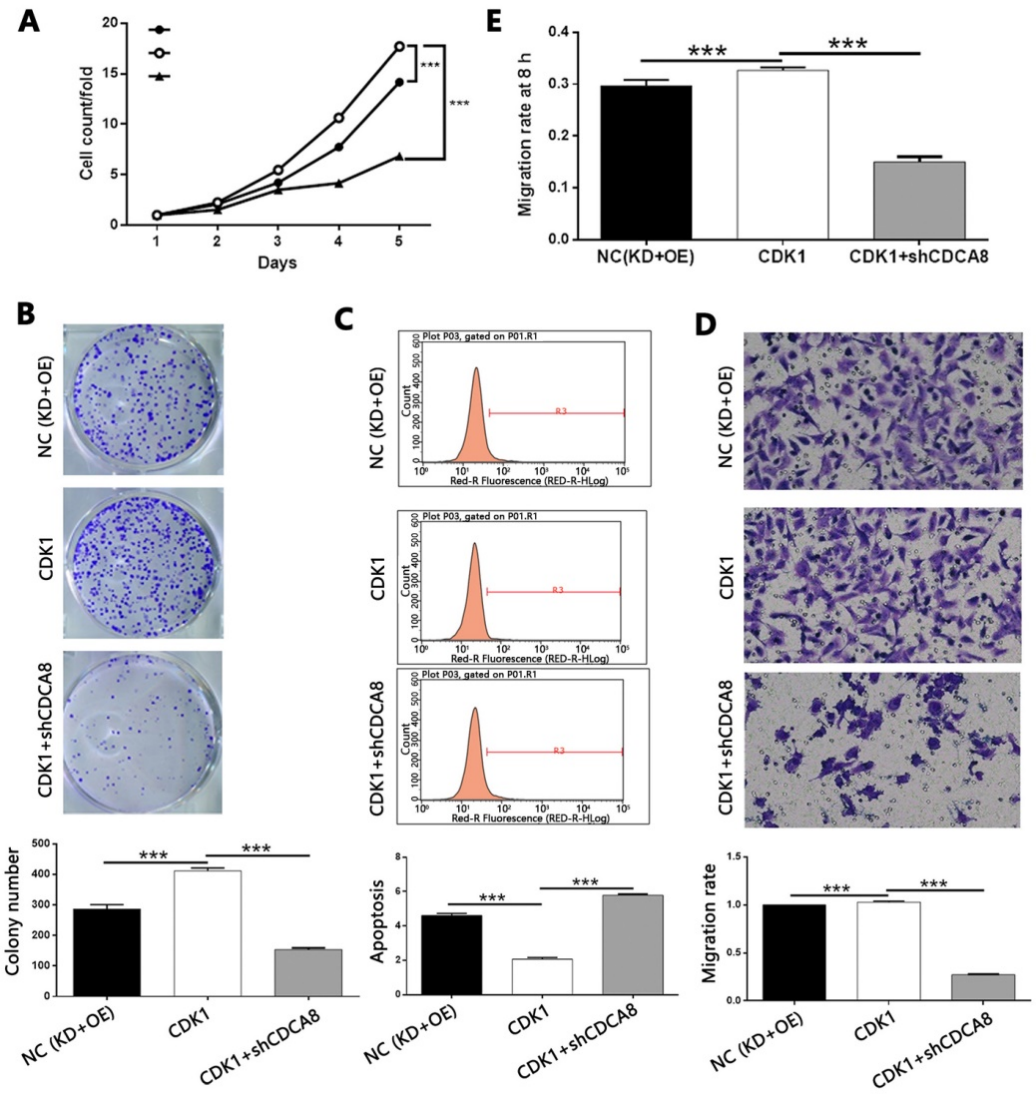
** Knockdown of CDCA8 impairs the promotion of thyroid cancer by CDK1 overexpression.** (A) CDCA8 knockdown attenuated the promotion of cell proliferation by CDK1 overexpression. (B) CDCA8 depletion inhibited the promoted colony formation induced by CDK1 overexpression. (C) CDCA8 knockdown reversed the inhibition of cell apoptosis by CDK1 overexpression. Outcomes of wound-healing assay (D) and Transwell assay (E) showed that CDCA8 downregulation impairs the promotion of cell migration induced by CDK1 overexpression. The representative image was randomly selected from at least 3 independent experiments. The data was shown as mean with SD. *P<0.05, **P<0.01, ***P<0.001

In order to explore the mechanism of CDCA8 regulating thyroid cancer, we constructed the CDCA8 knockdown and CDK1 overexpression TPC-1 cells, and compared with the results of only CDK1 overexpression cell model. After the transfection efficiency was confirmed by fluorescence imaging (Figure S8), the expression of CDCA8 and CDK1 was confirmed by qPCR and Western blotting (Figure S9). The following experiments were evaluated by the corresponding methods mentioned above, including the study of the synergistic effects of CDCA8 knockdown and CDK1 overexpression on cell proliferation (Figure 8A), colony formation (Figure 8B), apoptosis (Figure 8C) and cell migration (wound healing, Figure 8D; Trans-well test, Figure 8E). Interestingly, we found that the effect of CDK1 overexpression on the phenotype of TPC-1 cells could be significantly reduced or even reversed with the knockdown of CDCA8.

## Discussion

In this study, our work focused on exploring novel tumor promotor that possessed key functions in the development and progression of thyroid cancer. Through high-content screening of the inhibitory effects on cell proliferation by gene knockdown, CDCA8 was identified as the most promising candidate among the differentially expressed genes obtained by RNA-seq between thyroid cancer and corresponding normal tissues.

Chromosomal passenger complex is an important participant in cell proliferation and division, which consists of four components: INCENP, Survivin, Aurora B and CDCA8 12. As an important regulatory factor in mitosis, CDCA8 could assist in the localization of CPC centromere, correct kinetochore binding sites and stabilize bipolar spindle 11. The enhanced transcriptional activity of CDCA8 has been found in embryo, embryonic stem cells and cancer cells, while knockdown of CDCA8 can effectively inhibit the proliferation of lung cancer, colon cancer cells and human embryonic stem cells, and promote cell differentiation or induce differentiation 24, 25. Meanwhile, high expression of CDCA8 in gastric cancer, lung cancer and colon cancer were associated with poor prognosis of patients 26-29. Our results also found a negative correlation between CDCA8 expression with relapse free survival of patients. Recently, Jiao *et al.* surmised the correlation between CDCA8 and Forkhead Box M1 (FOXM1) in breast cancer through the combination of public microarray data analysis and immunohistochemistry staining of tumor tissues 30. Similarly, Bu *et al.* also found CDCA8 as a tumor promotor in estrogen-stimulated cell proliferation of breast cancer, which induced downregulation of CCND1 and Bcl-2, and upregulation of P21 and P27, and could predict poor prognosis 20. Besides, CDCA8 promoted the development and metastasis of cutaneous melanoma which was identified as a malignant tumor, and significant correlation between CDCA8 high expression and poorer prognosis of patients was also established 19. All the investigations highlighted the clinical value and significance of CDCA8 in cancer progression. Despite of these, to the best of our knowledge, the role in thyroid cancer played by CDCA8 has not been studied and remains largely unclear, which stimulates the performance of our study. Herein, CDCA8 obviously presented higher expression in thyroid cancer tissues than normal tissues. Downregulation of CDCA8 in thyroid cancer cells suppressed the cell or tumor growth of thyroid cancer *in vitro* or* in vivo*. Moreover, CDCA8 depletion also induced cell apoptosis and disturbed cell migration ability of thyroid cancer cells. All these results indicated that the development and progression of thyroid cancer could be possibly driven by CDCA8, and knockdown of which may be a promising strategy for the treatment of thyroid cancer.

A preliminary mechanism study was performed by detecting the effects of CDCA8 silencing on the expression of apoptosis related proteins or classical cancer related molecules. Among the various apoptosis related proteins, it was demonstrated that CD40/CD40L axis, which has been proved to be involved in a variety of diseases including cancer, was significantly upregulated in CDCA8-depleted cells 31, 32. Fas/FasL is also a typical axis that is capable of inducing cell apoptosis thus mediating the regulation of human cancers such as breast cancer and thyroid cancer 33, 34. Consistently, the upregulation of Fas and FasL in shCDCA8 group of cells was detected, by which may CDCA8 influence the cell apoptosis of thyroid cancer. Besides, the outcomes of the array analysis also revealed the upregulation of well-known pro-apoptotic proteins such as Bax and Caspase 3, and anti-apoptosis proteins such as Survivin and XIAP. Moreover, mass evidence showed the involvement of Akt phosphorylation in the development and progression of human cancers, including thyroid cancer 35, 36. In this study, the suppressed phosphorylation of Akt was also observed in cells with downregulated CDCA8. The expression of CCND1 and CDK4/6, which are well-acknowledged regulator not only in cell cycle but also in cancer progression, was also found to be downregulated upon CDCA8 knockdown 37-39.

In addition, this work further describes the investigation of downstream mechanism of CDCA8 regulating the progression of thyroid cancer. Function analysis indicated the participation of CDCA8 in M phase of mitotic cell cycle, nuclear division and chromosome segregation. It followed that CDCA8 was significantly associated with the biological process in terms of cell cycle. A variety of DEGs were obtained in CDCA8 knockdown cells through detecting and comparing the global gene expression profile of cells with or without CDCA8 knockdown. Finally, CDK1 was identified as the promising target of CDCA8 through combining the verification of most regulated DEGs and the involvement of 'Cyclin and cell cycle regulation' signalling pathway revealed by IPA enrichment analysis of canonical signalling pathway.

CDKs, which belong to the serine/threonine protein kinase family, have been reported to have 20 different family subtypes, all of which contain a homologous sequence of PSTAIRE, and through the binding of this sequence to the corresponding regulatory subunit - cyclin to form active heterodimers, which are involved in physiological processes such as transcription, metabolism, neural differentiation and development 40. Some members of CDK family including CDK1, CDK2, CDK4 and CDK6 have been reported to be involved in the regulation of cell cycle, thus participating the development and progression of cancer 41. Additionally, CDK1 has also been found to be vital mediator in the progress of thyroid cancer. Wu et al. suggested that CDK1 may serve as a novel diagnosis biomarker and potential therapeutic target for anaplastic thyroid cancer 42. Several studies also determined CDK1 as a key gene in anaplastic thyroid cancer by bioinformatics analysis 43-45. Zheng et al. revealed the overexpression of CDK1 in thyroid cancer based on multiple detection methods that combine independent cohorts 46. Interestingly, Lv et al. suggested that high iodine can induce the proliferation of thyroid cancer cells through AKT-mediated Wee1/CDK1 axis, providing a new insight into the regulation of proliferation of thyroid cancer cells by iodine 47. In addition, Xie *et al.* reported the possibility of CDK1 as a target of microRNA-424 thus mediating the regulation of thyroid 48. In their work, the ability of CDK1 knockdown arrested cell cycle in G2/M phase in agreement with its role in cell cycle regulation, and the inhibitory function of CDK1 silence on thyroid cancer cell proliferation was also observed in our study. Besides, we further demonstrated that the silenced or ectopic expression of CDK1 could induce inhibition or promotion of colony formation, cell mobility and the opposite effects on cell apoptosis. More importantly, all the regulatory effects induced by CDK1 overexpression could be alleviated or even reversed by CDCA8 knockdown, indicating its potential as the target of CDCA8 during regulating thyroid cancer.

Although this study was limited by several drawbacks such as the insufficient clinical samples and the not fully understanding of mechanism, the outcomes allow us to present the first report of the role played by CDCA8 in the development and progression of thyroid cancer. The oncogene-like functions of CDCA8 in thyroid cancer were clarified by its upregulation in thyroid cancer, and knockdown of which suppresses thyroid cancer development *in vitro* and *in vivo*. Moreover, CDK1 was identified as a potential target of CDCA8 for exerting its effects on thyroid cancer. Therefore, this work displays the promising potential of CDCA8 as a therapeutic target in the future development of molecular targeted drugs for thyroid cancer.

## Supplementary Material

Supplementary figures and table.

## Figures and Tables

**Table 1:** Expression patterns of CDCA8 in thyroid cancer tissues and normal tissues revealed in immunohistochemistry analysis

CDCA8 expression	Tumor tissue		Normal tissue		P-value
	Cases	Percentage	Cases	Percentage	
Low	17	42.5%	40	100%	*<*0.001
High	23	57.5%	0	0%	
Total	40	100.0%	40	100.0%	

**Table 2:** Relationship between CDCA8 expression and tumor characteristics in patients with thyroid cancer

Features	No. of patients	CDCA8 expression		*P* value
		low	high	
Age (years)				
<40	19	10	9	0.223
≥40	21	7	14	
Gender				
Male	8	4	4	0.636
Female	32	13	19	
T Infiltrate				
T1	2	1	1	0.563
T2	24	9	15	
T3	10	6	4	
T4	4	1	3	
Stage				
I	24	13	11	0.061
II	9	2	7	
III	3	1	2	
IV	4	0	4	

## Abbreviations

CDCA8: cell division cycle associated 8
CPC: Chromosomal passenger complex
Aurora B: Aurora kinase B
BIRC5: baculoviral IAP repeat containing 5
IHC: Immunohistochemistry
NIH: Image J software
Co-IP: Co-Immunoprecipitation
ECL: enhanced chemiluminescence
OD: optical density
