# Supplementary material for: CDCA8 Contributes to the Development and Progression of Thyroid Cancer through Regulating CDK1
Source: J Cancer. 2022 Apr 18;13(7):2322–35. doi: 10.7150/jca.64747 (PMC9066215; doi:10.7150/jca.64747)
Supplement: Supplementary file 1 — Supplementary figures and table. [file jcav13p2322s1.pdf]

Table S1 Antibodies used in western blotting and IHC

Primary antibodies	Dilution in WB	Source species	Company	Catalog No.
CDCA8	1:1000	Rabbit	abcam	ab70910
GAPDH	1:3000	Rabbit	Bioworld	AP0063
BIRC5	1:1000	Rabbit	abcam	ab469
CDK1	1:2000	Rabbit	abcam	ab133327
E2F1	1:1500	Rabbit	abcam	ab179445
PLK1	1:1000	Mouse	abcam	ab17057
SKP2	1:1000	Rabbit	abcam	ab183039
DYKDDDDK Tag*	1:50/1:1000	Rabbit	CST	14793

*Notes: DYKDDDDK Tag binds to same epitope as Sigma's Anti-FLAG® M2 Antibody

Primary antibodies	Dilution in IHC	Source species	Company	Catalog No.
CDCA8	1:30	Rabbit	abcam	ab70910
Ki67	1:200	Rabbit	abcam	ab16667
CDK1	1:100	Rabbit	sigma	HPA003387

Secondary antibody	Dilution		Company	Catalog No.
HRP Goat Anti-Rabbit IgG (WB)	1:3000		Beyotime	A0208
HRP Goat Anti-Mouse IgG (WB)	1:3000		Beyotime	A0216

HRP Goat Anti-Rabbit IgG (IHC)	1:200	Abcam	Ab111909

Table S2 The target sequences and shRNA sequences

Gene	No.	Target sequence (5'-3')	shRNA sequences (5'-3')
CDCA8	Pbr10539-a	GTGGAAATACGAATCAAGCAA	CcggGTGGAAATACGAATCAAGCAActcgagTTGCTTGATTCGTATTTCCACTTTTTg
	Pbr10539-b	GTGGAAATACGAATCAAGCAA	aattcaaaaaGTGGAAATACGAATCAAGCAActcgagTTGCTTGATTCGTATTTCCAC
CDCA8	Pbr10540-a	GTGGAAATACGAATCAAGCAA	ccggAGATGAAATGATAGTGGAAGActcgagTCTTCCACTATCATTTCATCTtttttg
	Pbr10540-b	GTGGAAATACGAATCAAGCAA	aattcaaaaaAGATGAAATGATAGTGGAAGActcgagTCTTCCACTATCATTTCATCT
CDCA8	Pbr10541-a	GCGGAGAGAGCCTGCGATTAT	CcggGCGGAGAGAGCCTGCGATTATctcgagATAATCGCAGGCTCTCTCCGCTTTTTg
	Pbr10541-b	GCGGAGAGAGCCTGCGATTAT	aattcaaaaaGCGGAGAGAGCCTGCGATTATctcgagATAATCGCAGGCTCTCTCCGC
CDK1	Pbr10545-a	TTCCATGGATCTGAAGAAATA	CcggTTCCATGGATCTGAAGAAATActcgagTATTTCTTCAGATCCATGGAATTTTTg
	Pbr10545-b	TTCCATGGATCTGAAGAAATA	aattcaaaaaTTCCATGGATCTGAAGAAATActcgagTATTTCTTCAGATCCATGGAA
CDK1	Pbr10546-a	AGACTAGAAAGTGAAGAGGAA	CcggAGACTAGAAAGTGAAGAGGAActcgagTTCCTCTTCACTTTCTAGTCTTTTTTg
	Pbr10546-b	AGACTAGAAAGTGAAGAGGAA	aattcaaaaaAGACTAGAAAGTGAAGAGGAActcgagTTCCTCTTCACTTTCTAGTCT
CDK1	Pbr10547-a	ATGGAGTTGTGTATAAGGGTA	CcggATGGAGTTGTGTATAAGGGTActcgagTACCCTTATACACAACTCCATTTTTTg
	Pbr10547-b	ATGGAGTTGTGTATAAGGGTA	aattcaaaaaATGGAGTTGTGTATAAGGGTActcgagTACCCTTATACACAACTCCAT

Table S3 Primers used in qPCR

Gene	Forward primer sequence (5’-3’)	Reverse primer sequence (5’-3’)
ARL6IP1	ATGGGGAGAAGTGATGCTGATG	CGCTAGAATGGGAACAAGGTAGTC
CDC20	AATGGAGCAGCCTGGGGAATA	CGGGCAGAGTGACTGGTCATAT
GAS2L3	CCCAGCGTGCCAGTTAGTATTC	GCCTGCGGTTTCTTCGTTATG
NEK2	GAAGGAATGCCACAGACGAAGT	CAAGCAGCCCAATGACCAGATA
KIF20A	GGCCGTTCCTGCATGATTGT	TGTCTGCCTTAGCCCCTTTCT
HMMR	TTAACAAGTGGCGTCTCCTCTATG	CTGAGCTGCACCATGTTCATTC
CDCA8	AGACAGGCAGAACCTCCTCAAG	TTCCTCCAAGGGCGAAGTAGTC
CDK1	TCAGGATGTGCTTATGCAGGATTC	TCCATGTACTGACCAGGAGGGA
POLQ	TCCTACACCCATTCCAACATCTG	GTCTTTGAACCCATTTCTACTCCC
GBP2	TGATGACTACTTGGAGCTTTCGC	GGAATGGCTGAGGATGTAGGAAC
GAPDH	TGACTTCAACAGCGACACCCA	CACCCTGTTGCTGTAGCCAAA
FANCD2	GGCTTGACAGAGTTGTGGATG	TAGGATCTCAGGTAGGCTGGTG
CDC25A	TGGAAGTACAAAGAGGAGGAAGAG	GCCAGGGATAAAGACTGATGAAG
CDCA3	CACGGACACCTATGAAGACCA	GTTTGGAGGGGAACTCAGTCT
PLK1	AGAGGAGGAAAGCCCTGACT	TAACTCGGTTTCGGTGCAGG

BIRC5	TCTCAAGGACCACCGCATCT	TTTGCATGGGGTCGTCATCT
SFRP1	CGTGGGCTACAAGAAGATGG	GAAGAGCGAGCAGAGGAAGA
FOXM1	AGTTCCCGGTGAACCAGTCA	ACACCACCTGTTCCCCAAA
CDK1	TCAGGATGTGCTTATGCAGGATTC	TCCATGTACTGACCAGGAGGGA
KIF2C	AGATGGAAGCCTGCTCTAACGG	GCTGGACATCTGGGAAGACAGT
BRCA1	TGGCAACATACCATCTTCAACC	TGTCAATTCTGGCTTCTCCCT
CCNE2	AGCTGGTCTGGCGAGGTTTT	GGCCTGGATTATCTGGGCTTC
AURKB	CATGGAGGAGTTGGCAGATG	CCTTGAGCCCTAAGAGCAGAT
CDK2	ATCGCAAATGCTGCACTACG	GGTCACATCCTGGAAGAAAGG
CHEK1	TTGGCTTGGCAACAGTATTTCG	CCAGCGAGCATTGCAGTAAGT
LEF1	AATAAAGTGCCCGTGGTGC	GAGAAAAGTGCTCGTCACTGTAAG
CBX5	CTTCAGAGGATGAGGAGGAGTATG	GCTCAGGGCAATCCAAGTTTT
SKP2	ATAGAAGTGTCCACCCTCCACG	CACCCAGAAAGGTTAAGTCGC
E2F1	CACTTTCGGCCCTTTTGCTC	GTGCTCTCACCGTCCTACAC
RFC3	TGATGCTGGAAATAGTGACCG	CGCCAAGCACCTACTACGAATA
RRM1	GAAAGAGCAACCAGCAGAACC	GGGAAGCCAAATTACAAACAGC

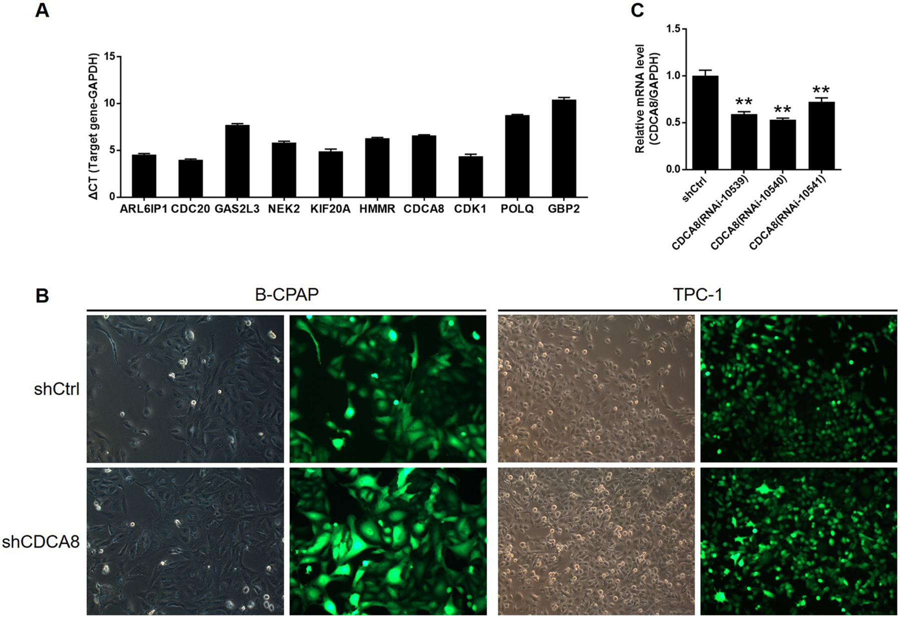

A
ΔCT (Target gene-GAPDH)
15
10
5
0
ARL6IP1
CDC20
GAS2L3
NEK2
KIF20A
HMMR
CDCA8
CDK1
POLQ
GBP2
C
Relative mRNA level (CDCA8/GAPDH)
1.5
1.0
0.5
0.0
**
**
**
shCtrl
CDCA8(RNAi-10539)
CDCA8(RNAi-10540)
CDCA8(RNAi-10541)
B
B-CPAP
TPC-1
shCtrl
shCDCA8

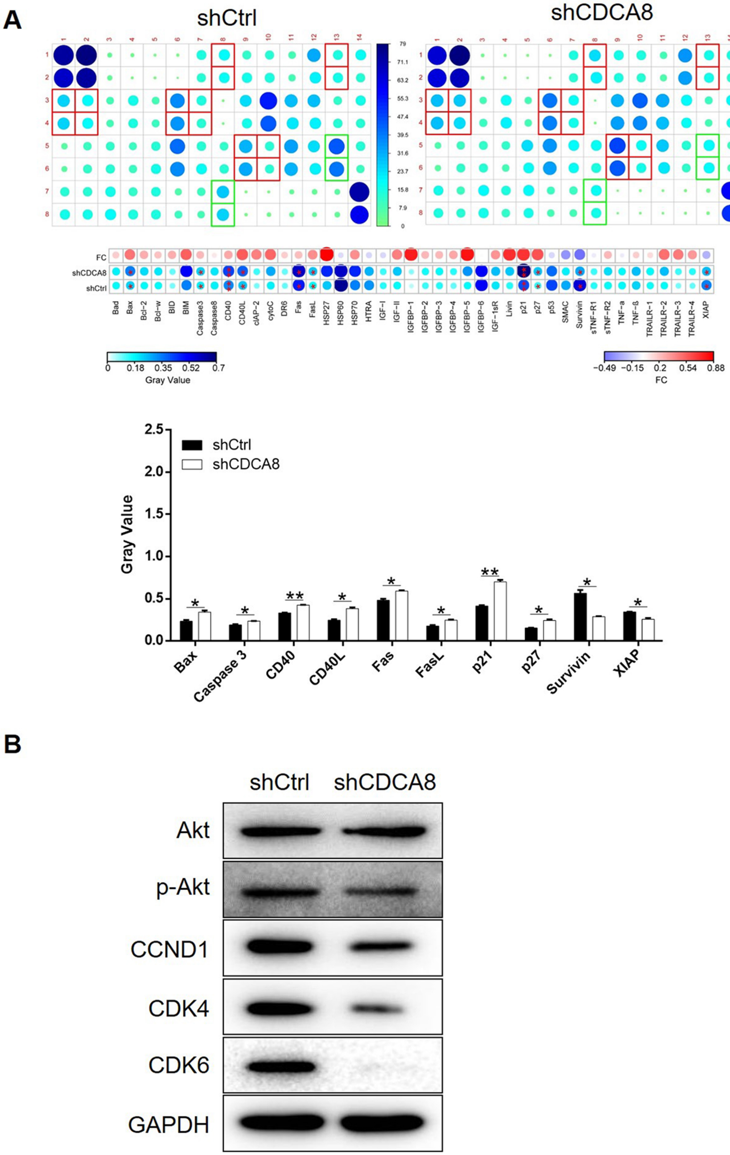
A
shCtrl
shCDCA8
FC
shCDCA8
shCtrl
Bad
Bax
Bcl-2
Bcl-w
BID
BIM
Caspase3
Caspase8
CD40
CD40L
cIAP-2
cytoC
DR6
Fas
FasL
HSP27
HSP60
HSP70
HTRA
IGF-I
IGF-II
IGFBP-1
IGFBP-2
IGFBP-3
IGFBP-4
IGFBP-5
IGFBP-6
IGF-1sR
Livin
p21
p27
p53
SMAC
Survivin
sTNF-R1
sTNF-R2
TNF-a
TNF-ß
TRAILR-1
TRAILR-2
TRAILR-3
TRAILR-4
XIAP
Gray Value
FC
shCtrl
shCDCA8
Gray Value
Bax
Caspase 3
CD40
CD40L
Fas
FasL
p21
p27
Survivin
XIAP
B
shCtrl
shCDCA8
Akt
p-Akt
CCND1
CDK4
CDK6
GAPDH

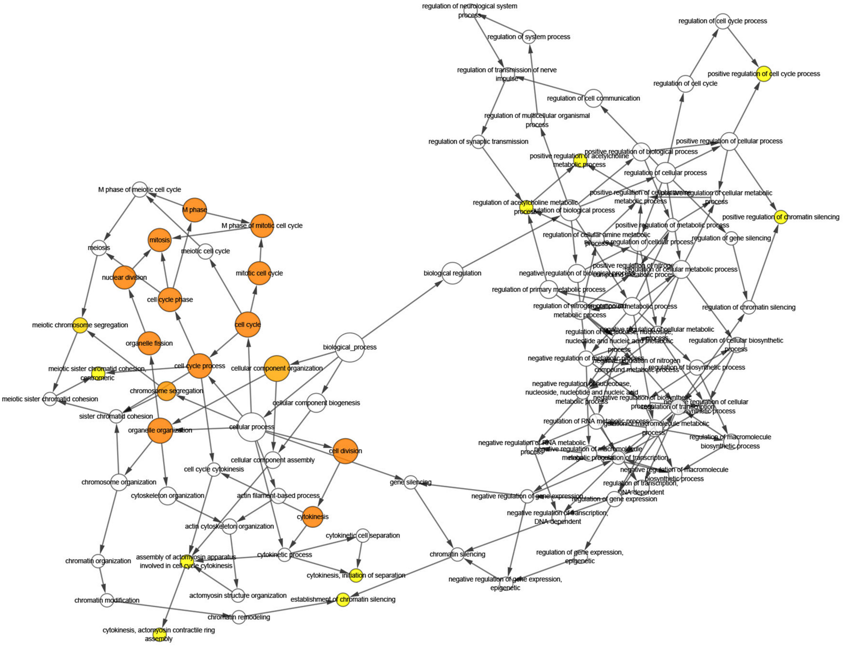
regulation of neurological system process
regulation of system process
regulation of cell cycle process
regulation of transmission of nerve impulse
positive regulation of cell cycle process
regulation of cell cycle
regulation of cell communication
regulation of multicellular organismal process
regulation of synaptic transmission
positive regulation of cellular process
positive regulation of biological process
positive regulation of acetylcholine metabolic process
regulation of cellular process
M phase of meiotic cell cycle
positive regulation of cellular metabolic process
regulation of acetylcholine metabolic process
M phase
M phase of mitotic cell cycle
positive regulation of chromatin silencing
positive regulation of metabolic process
mitosis
meiosis
meiotic cell cycle
regulation of cellular amine metabolic process
regulation of gene silencing
mitotic cell cycle
nuclear division
biological regulation
negative regulation of biological process
regulation of cellular metabolic process
regulation of primary metabolic process
cell cycle phase
regulation of chromatin silencing
regulation of metabolic process
meiotic chromosome segregation
cell cycle
organelle fission
biological_process
regulation of cellular biosynthetic process
negative regulation of metabolic process
meiotic sister chromatid cohesion, centromeric
cell cycle process
cellular component organization
regulation of biosynthetic process
chromosome segregation
negative regulation of nucleobase, nucleoside, nucleotide and nucleic acid metabolic process
meiotic sister chromatid cohesion
cellular component biogenesis
sister chromatid cohesion
regulation of RNA metabolic process
organelle organization
cellular process
regulation of macromolecule biosynthetic process
negative regulation of RNA metabolic process
cell division
cellular component assembly
cell cycle cytokinesis
negative regulation of macromolecule biosynthetic process
chromosome organization
gene silencing
cytoskeleton organization
actin filament-based process
negative regulation of gene expression
regulation of transcription, DNA-dependent
regulation of gene expression
cytokinesis
negative regulation of transcription, DNA-dependent
actin cytoskeleton organization
cytokinetic cell separation
regulation of gene expression, epigenetic
chromatin organization
assembly of actomyosin apparatus involved in cell cycle cytokinesis
cytokinetic process
chromatin silencing
cytokinesis, initiation of separation
negative regulation of gene expression, epigenetic
chromatin modification
actomyosin structure organization
establishment of chromatin silencing
chromatin remodeling
cytokinesis, actomyosin contractile ring assembly

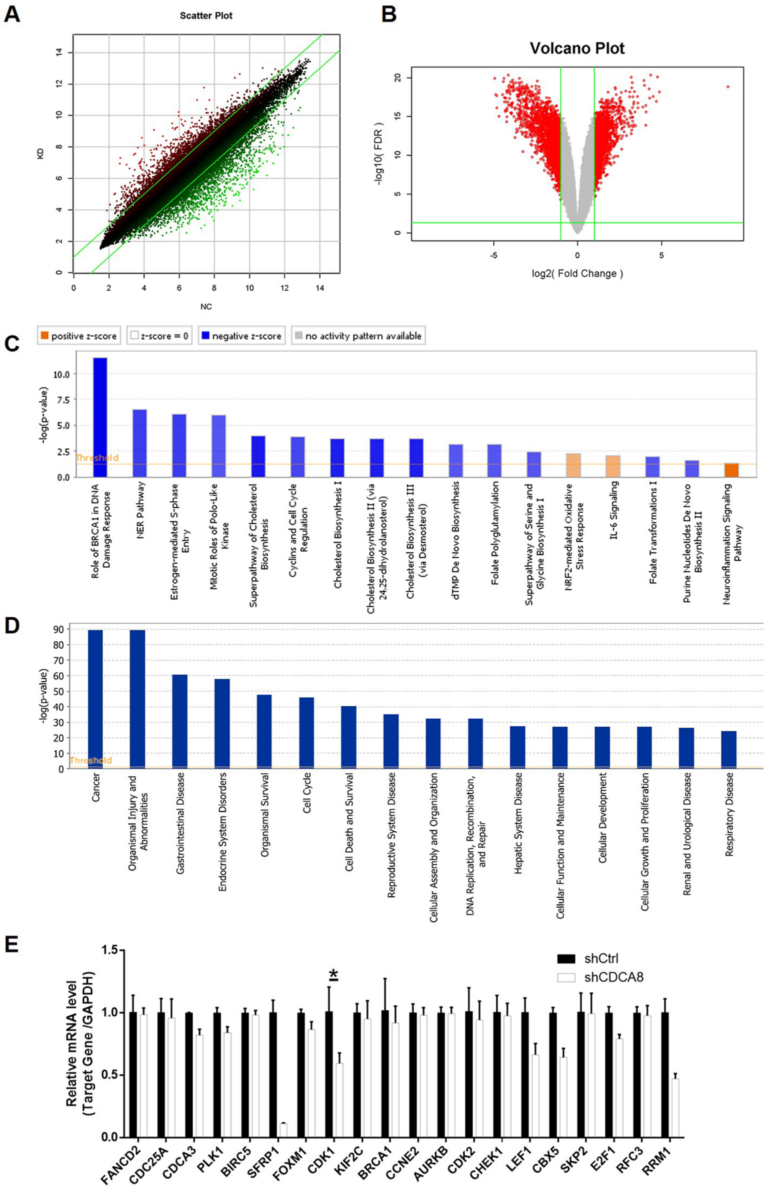
A
Scatter Plot
KD
NC
B
Volcano Plot
-log10(FDR)
log2(Fold Change)
C
positive z-score
z-score = 0
negative z-score
no activity pattern available
-log(p-value)
Threshold
Role of BRCA1 in DNA Damage Response
NER Pathway
Estrogen-mediated S-phase Entry
Mitotic Roles of Polo-Like Kinase
Superpathway of Cholesterol Biosynthesis
Cyclins and Cell Cycle Regulation
Cholesterol Biosynthesis I
Cholesterol Biosynthesis II (via 24,25-dihydrolanosterol)
Cholesterol Biosynthesis III (via Desmosterol)
dTMP De Novo Biosynthesis
Folate Polyglutamylation
Superpathway of Serine and Glycine Biosynthesis I
NRF2-mediated Oxidative Stress Response
IL-6 Signaling
Folate Transformations I
Purine Nucleotides De Novo Biosynthesis II
Neuroinflammation Signaling Pathway
D
-log(p-value)
Threshold
Cancer
Organismal Injury and Abnormalities
Gastrointestinal Disease
Endocrine System Disorders
Organismal Survival
Cell Cycle
Cell Death and Survival
Reproductive System Disease
Cellular Assembly and Organization
DNA Replication, Recombination, and Repair
Hepatic System Disease
Cellular Function and Maintenance
Cellular Development
Cellular Growth and Proliferation
Renal and Urological Disease
Respiratory Disease
E
Relative mRNA level (Target Gene /GAPDH)
shCtrl
shCDCA8
*
FANCD2
CDC25A
CDCA3
PLK1
BIRC5
SFRP1
FOXM1
CDK1
KIF2C
BRCA1
CCNE2
AURKB
CDK2
CHEK1
LEF1
CBX5
SKP2
E2F1
RFC3
RRM1

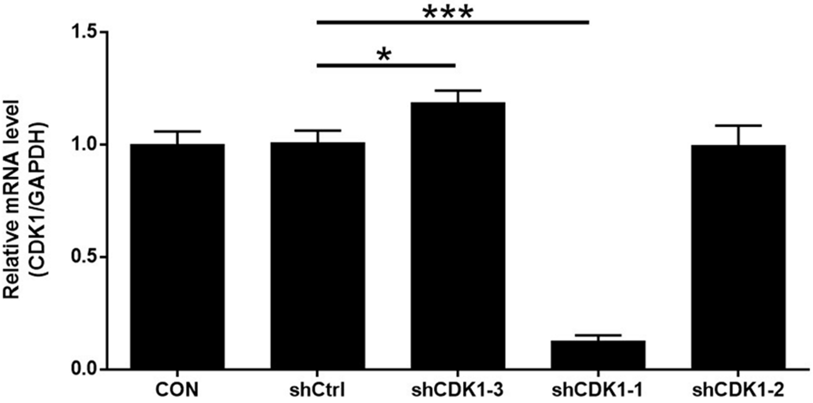
***
*
1.5
1.0
0.5
0.0
Relative mRNA level
(CDK1/GAPDH)
CON
shCtrl
shCDK1-3
shCDK1-1
shCDK1-2

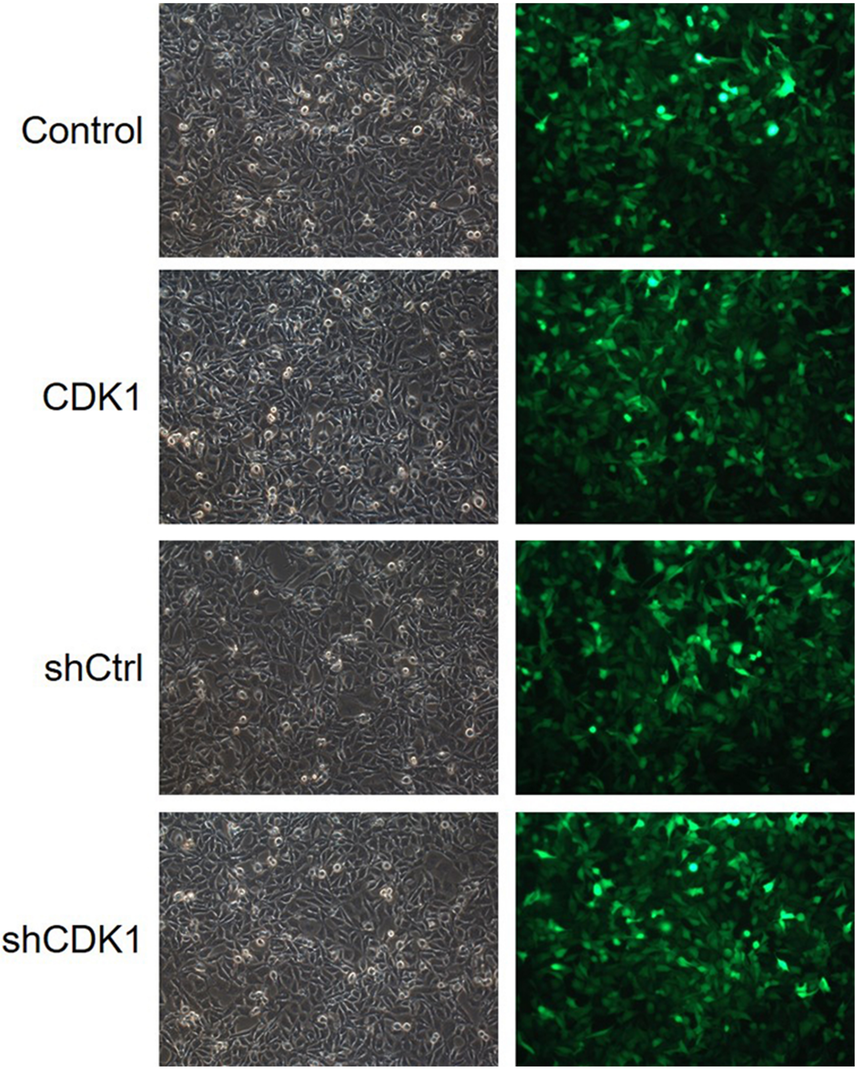
Control
CDK1
shCtrl
shCDK1

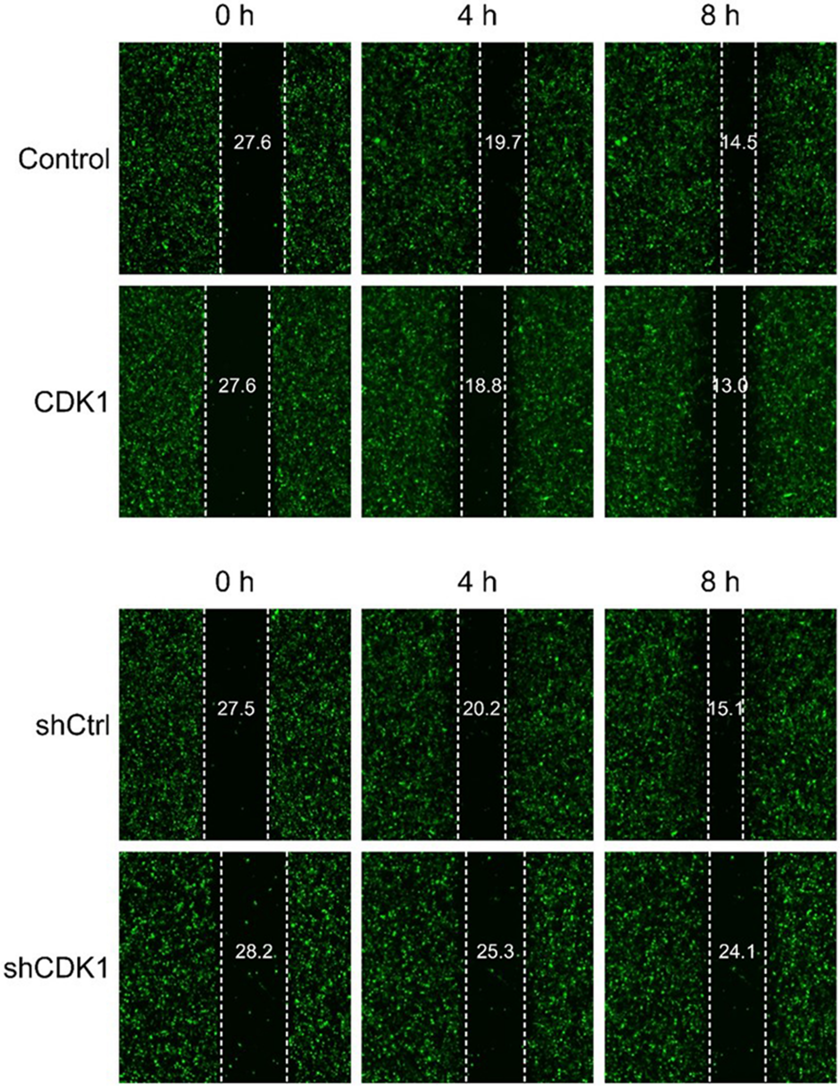
0 h
4 h
8 h
Control
27.6
19.7
14.5
CDK1
27.6
18.8
13.0
0 h
4 h
8 h
shCtrl
27.5
20.2
15.1
shCDK1
28.2
25.3
24.1

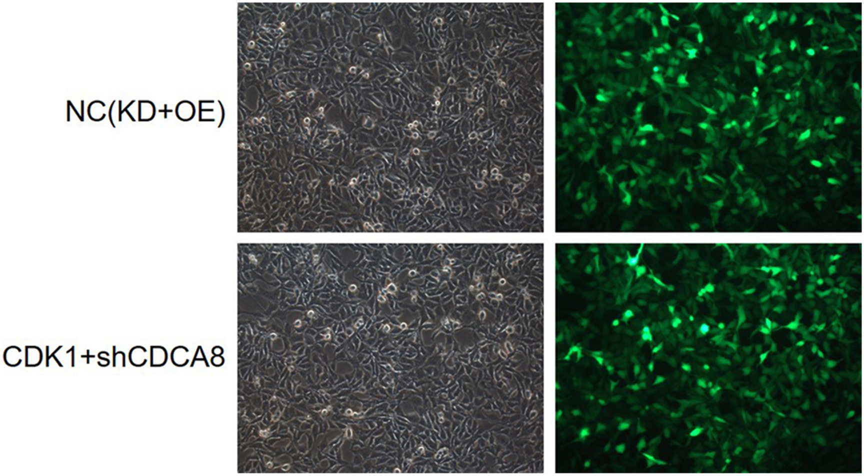
NC(KD+OE)
CDK1+shCDCA8
